# Benchmarking hydration, navel health, and transfer of passive immunity in surplus dairy calves

**DOI:** 10.3168/jdsc.2024-0693

**Published:** 2025-02-20

**Authors:** Ting-Yu Cheng, Jessica A. Pempek, David L. Renaud, Kathryn L. Proudfoot, Zachary England, Devon J. Wilson, Gregory Habing

**Affiliations:** 1 Department of Veterinary Preventive Medicine, College of Veterinary Medicine, The Ohio State University, Columbus, OH 43210; 2 USDA-Agricultural Research Service (ARS) Livestock Behavior Research Unit, West Lafayette, IN 47907; 3 Department of Population Medicine, University of Guelph, Guelph, ON, Canada, N1G 2W1; 4 Sir James Dunn Animal Welfare Centre, Atlantic Veterinary College, University of Prince Edward Island, Charlottetown, PE, Canada, C1A 4P3; 5 Department of Animal Sciences, College of Food, Agricultural, and Environmental Sciences, The Ohio State University, Columbus, OH 43210

## Abstract

Surplus dairy calves, or calves that are not used to replace the milking herd, are often sold within the first week of life and can sometimes receive suboptimal early-life care. The aim of this study was to assess the effect of delivering benchmarking reports to source dairy farms on the health of subsequent surplus calf cohorts. Briefly, 10 farms were first blocked by herd size category, and within each block, farms were alternately assigned to intervention and control in descending order of the previously measured proportion of calves with adequate passive transfer immunity (i.e., ≥5.8 g/dL total serum protein). After the initial randomization, 3 additional farms were enrolled by convenience to increase the statistical power, and one farm was assigned to the intervention group. Overall, 13 dairy farms were recruited and assigned to intervention (n = 6) and control (n = 7) groups. The intervention group received health benchmarking reports of surplus calves delivered to calf dealers halfway through the study period, whereas the control group received no reports. Between May 2021 and June 2022, a total of 282 and 371 calves enrolled from intervention and control farms, respectively, were assessed for hydration, navel health, and transfer of passive immunity (TPI). In brief, hydration was evaluated using the skin tent test and a 4-point scale, and navel health was assessed based on the width of umbilical cords using a 4-point scale. The TPI was assessed based on the concentration of total serum protein. In November 2021, benchmarking reports containing health metrics of calves from 13 farms during the first 6 mo of the study were delivered to 6 intervention farms. Differences in health metrics of subsequent surplus calf cohorts between intervention and control farms were investigated using generalized linear mixed models specified to recognize “farm” as the experimental unit. Prevalence of dehydration was decreased in calves from intervention farms compared with control farms after receiving the benchmarking reports (odds ratio = 0.19, 95% CI = [0.04, 0.90] %). There was no evidence of any differences in navel inflammation or failure in TPI associated with benchmarking reports. Results suggest delivering benchmarking reports to dairy producers may improve hydration in surplus calves. Nevertheless, additional methods should be investigated to improve calf health within the surplus calf production chain.

## Highlights

• Delivering calf health benchmarking reports reduced surplus calf dehydration.
• No significant effects in navel inflammation and failed transfer of passive immunity.
• Providing calf post-marketing performance may improve on-farm calf care practice.

## Graphical Abstract

**Figure:**
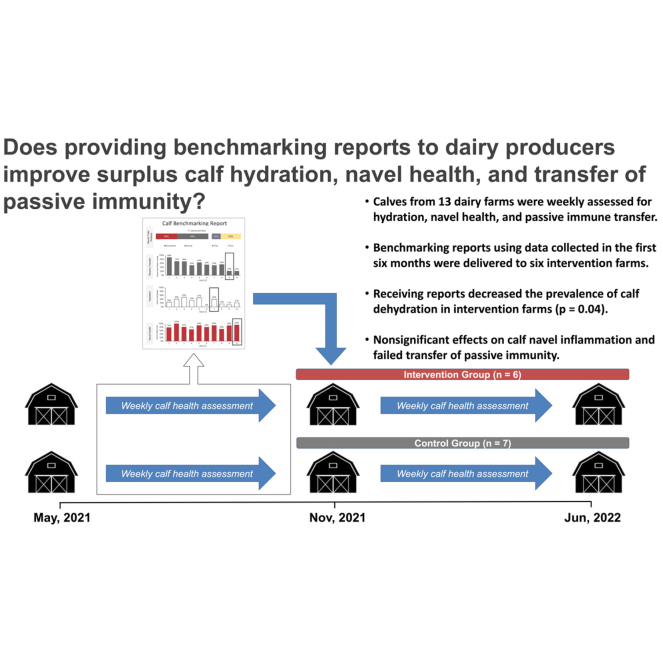
**Summary:** More than half of the dairy calf population is sold soon after birth to the veal or dairy-beef industries as surplus calves. Producers often receive little feedback on the health of surplus calves after their sale. This study investigated the impact of producers receiving benchmarking reports on hydration, navel health, and transfer of passive immunity after the sale of surplus calves on the health of subsequent surplus calves on their farms. We found that benchmarking calf health was associated with a reduction in prevalence of surplus calf dehydration, but there was no evidence of any additional effects on health and transfer of passive immunity.

The importance of neonatal calf care on the dairy farm of birth has been well documented to prevent calf morbidity and mortality (Godden et al., 2019). However, evidence has shown that male and nonreplacement female dairy calves are perceived to have a lower value compared with their female counterparts (Wilson et al., 2021) and thereby sometimes receive suboptimal care and are sold within the first week of life (Cheng et al., 2024). Recent estimates revealed a high prevalence of dehydration, navel inflammation, and failed transfer of passive immunity (**FTPI**) as common clinical concerns in surplus calves (Pempek et al., 2017; Renaud et al., 2018; Maggard et al., 2024), underscoring the need to investigate strategies to promote health and welfare of surplus calves before departure from source dairy farms.

Benchmarking is an effective strategy to promote the learning, exchange of ideas, and adoption of best practices by comparing one's performance to peers (Atkinson et al., 2017; Sumner et al., 2018), and has been documented to improve animal health (e.g., lameness, hock lesion, FTPI) in dairy production (Chapinal et al., 2014; Atkinson et al., 2017). Because dairy producers rarely receive post-sale feedback on their surplus calves (Cheng et al., 2024), this study sought opportunities to improve calf health by testing the hypothesis that providing benchmarking reports to dairy producers would improve surplus calf hydration, navel health, and transfer of passive immunity (**TPI**).

Data collection was conducted as a continuation of a previously reported cross-sectional study of calf health at 2 calf dealers (A and B; Maggard et al., 2024).

The dairy farms (n = 13) that had delivered the most calves to 2 calf dealers during the preceding cross-sectional study (May–September 2021) and were located within a 3-h drive from The Ohio State University (**OSU**) main campus (Columbus, OH) were initially contacted via phone calls by the research team (JP and GH). In several instances, the research team had only the farm addresses and made the initial contact by visiting the farm. Ten farms consented to participate in the study, have the research team assess their calves, and receive benchmarking reports of calf health if assigned to the intervention group. After the completion of the previous cross-sectional study, enrolled farms were assigned to the intervention and control groups using a stratified randomization approach. In brief, farms were first blocked by herd size based on the median number of milking cows (i.e., above or below the median herd size across farms of 460 cows). Within each block, farms were sorted in descending order of the proportion of calves (assessed between May and September 2021; Maggard et al., 2024) with ≥5.8 g/dL total serum protein (**TSP**; i.e., “good” TPI; Lombard et al., 2020). Thereafter, farms were assigned to the intervention (n = 5 farms) or control (n = 5 farms) group alternatively (farms 1–10, Table 1). Because farm and calf enrollment progressed slower than expected, 3 farms that had delivered the 11th to 13th most calves to 2 calf dealers were additionally enrolled after the initial group assignment (September–November 2021) to increase the statistical power. Among them, one (farm 11, Table 1) agreed to receive benchmarking reports and was assigned to the intervention group, whereas the other 2 (farms 12 and 13, Table 1) did not respond to the research team and were assigned to the control group. Overall, 6 and 7 farms were assigned to the intervention and control groups, respectively.

**Table 1:** Characteristics of dairy farms enrolled in this study and details of the experimental design

Farm ID	Herd size (no. of milking cows)	No. of calves assessed		Calf dealer	Calves with ≥5.8 g/dL total serum protein,^1^ % (n/n)	Treatment group^2^
		Pre-intervention	Post-intervention			
1	620	6	14	B	50.0 (3/6)	Control
2	850	15	15	A	53.3 (8/15)	Intervention
3	1,050	13	0	B	53.9 (7/13)	Control
4	700	78	77	A	63.8 (37/58)	Intervention
5	6,000	37	224	B	89.2 (33/37)	Control
6	220	14	7	A	21.4 (3/14)	Intervention
7	300	10	8	A	22.2 (2/9)	Control
8	130	10	22	A	50.0 (5/10)	Intervention
9	250	20	16	A	68.8 (11/16)	Control
10	220	12	6	A	72.7 (8/11)	Intervention
11	220	7	19	A	57.1 (4/7)	Intervention
12	—	12	1	A	45.5 (5/11)	Control
13	—	7	3	A	57.1 (4/7)	Control

1 Calculated based on calves assessed between May and September 2021 for a previous cross-sectional study (Maggard et al., 2024).

2 Farms 1 to 10 were stratified by herd size and alternately assigned to the treatment groups in descending order of the proportion of calves with ≥5.8 g/dL total serum protein. Farms 11 to 13 were assigned based on producers' willingness to receive the benchmarking report.

During the period of study, 6 intervention and 4 control farms delivered surplus calves to calf dealer A, whereas 3 control farms delivered calves to calf dealer B. Farms were located within 80 km from corresponding calf dealers, and both calf dealers were approximately 180 km from the OSU campus. Calf dealer A housed calves with sawdust bedding in group pens. Calves delivered to calf dealer A were sold on the same day without receiving milk replacer. Calf dealer B housed calves in group pens with straw bedding and varied amounts of water in troughs. However, due to the age of the calves and their inexperience in drinking from water troughs, little to no water intake occurred. Therefore, calves from enrolled farms were considered to have not received milk or water before the health assessments. The calf facilities at both dealers were naturally ventilated.

Calf dealers were visited 2 to 3 times per week between May 2021 and June 2022 by research personnel for calf health assessment (Figure 1). Given the nature of dairy production, calves were typically younger than 7 d of age at the time of sale and assessment (unknown actual age). At each visit, all available calves from enrolled farms were assessed within 6 h of arrival. The source dairy farm of calves was identified using calf ear tags and sales logs provided by the calf dealers. Calf hydration and navel health were assessed using a scoring system adapted from Maggard et al. (2024). Briefly, hydration was assessed using the skin tent test and a 4-point scale (Garcia et al., 2022), and navel health was assessed based on the width of umbilical cords using a 4-point scale (Pempek et al., 2017). Interrater reliability was measured before the start of the study, and the 2 examiners for the study had >92% reliability for the measures (Maggard et al., 2024). In addition to health assessment, one blood sample per calf was collected from the jugular vein using a 20-gauge needle (Becton Dickinson) and 12.5-mL serum separator tube (Becton Dickinson). Serum was harvested following centrifugation (1,000 × *g* for 15 min at room temperature) and loaded to a digital refractometer (Digital-Dairy refractometer, MISCO) for TSP measurement. The calf dealers and sample collectors were blinded from the intervention assignment.

**Figure 1:**
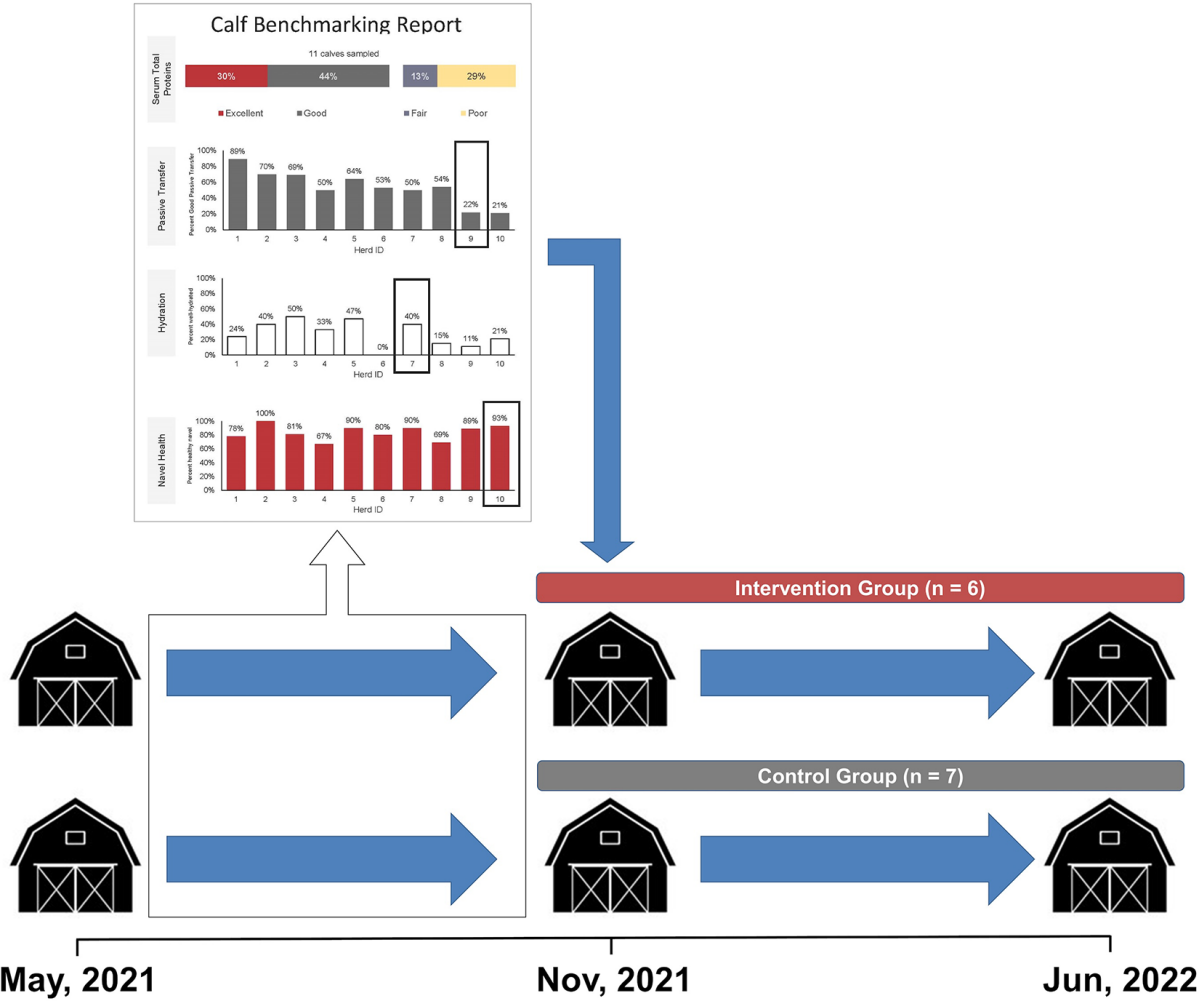
Schematic diagram of experimental design. Dairy calves from 13 dairy farms were assessed for dehydration, navel inflammation, and failed transfer of passive immunity at 2 calf dealers starting in May 2021. Data collected from May to November 2021 were summarized in calf health benchmarking reports (Supplemental File S1, see Notes) and delivered to the intervention farms. Calf health assessments at calf dealers continued until June 2022 to investigate the effect of benchmarking report delivery on calf health metrics.

In November 2021, farm-specific benchmarking reports were generated using data collected from May 2021 up to the date of report generation (i.e., May to November 2021, Figure 1). Reports contained (1) the number of calves evaluated and the proportion of calves with poor (TSP <5.1 g/dL), fair (TSP = 5.1–5.7 g/dL), good (TSP = 5.8–6.1 g/dL), and excellent (TSP >6.1 g/dL) TPI (Lombard et al., 2020); (2) the proportion of calves with TSP ≥5.1 g/dL; (3) the proportion of calves with adequate hydration (score = 0); and (4) the proportion of calves with good navel health (score = 0). De-identified summaries of the 3 health metrics from the remaining 12 farms were also included (dummy report available in Supplemental File S1, see Notes). A single report was delivered to producers in the intervention group through individualized in-person meetings (45 min to 1 h) with the research team (JP and GH). During the meetings, the research team explicitly explained the content of the report and the ranking relative to other farms, and encouraged the dairy producer to ask questions. Benchmarking reports were not delivered to control farms. After report delivery, calves from intervention and control farms continued to be assessed at the calf dealers for an additional 6 mo to investigate subsequent changes in health metrics.

Calf health data were digitized in an Excel (Microsoft Corp., Redmond, WA) spreadsheet, anonymized by randomly assigning numbers 1 to 13 to farms (Table 1), manually screened for input errors, and imported into SAS (SAS v. 9.4, SAS Institute, Cary, NC) for descriptive and exploratory analyses. The data that support the findings of this study are available from the corresponding author (GH) upon reasonable request.

Calf health data collected before the delivery of benchmarking reports (i.e., the first 6 mo of the study) were compiled separately for control and intervention farms to generate the baseline. Calves that received a dehydration or navel inflammation score of 1 to 3 were considered positive for corresponding signs, whereas those that received a score of 0 were labeled as negative. For FTPI, TSP concentrations were dichotomized using a cutoff of 5.1 g/dL (TSP ≥5.1 g/dL: FTPI absent; TSP <5.1 g/dL: FTPI present; Lombard et al., 2020).

Each binary response variable was fitted with a generalized linear mixed model assuming a Bernoulli distribution with a logit link function, along with the reception of benchmarking report (yes/no), period of sample collection (before/after report delivery), and their interaction term as fixed effects. In addition, whether the effect of the intervention was dependent on the pre-intervention calf health was investigated by including an interaction term between the intervention (received/did not receive benchmarking reports) and the proportion of calves with ≥5.8 g/dL TSP pre-intervention (Table 1) in each model. The model also included a random effect of the source dairy farm nested within the intervention and its cross-product with the period of sample collection relative to report delivery, to properly identify the experimental unit (source dairy farms) for each of the fixed effect factors, as separate from the individual calves serving as subsamples and units of observation. There was no evidence of overdispersion for all models based on the maximum-likelihood-based Pearson chi-squared statistic over degrees of freedom statistics. The final models were fitted using residual pseudo-likelihood as a method of estimation, with the degrees of freedom and standard error corrections estimated using the Kenward-Roger approximation (Kenward and Roger, 1997). Results were reported as estimated odds ratios (**OR**) of a calf having dehydration, navel inflammation, or FTPI at the calf dealer from intervention versus control farms and before versus following the delivery of benchmarking reports. Multiple comparisons were adjusted using a Bonferroni procedure to prevent inflation of the type I error. Additionally, model-based estimates with corresponding 95% confidence intervals were reported.

Table 1 shows general descriptives of the enrolled dairy farms. The median farm size was 460 lactating cows (130–6,000 cows). Overall, a total of 653 calves were assessed from the 6 intervention (n = 282) and 7 control farms (n = 371). The sex was recorded for 551 calves (84.4%, 551/653), where 419 (76.0%, 419/551) were male and 132 were female (24.0%, 132/551). Additionally, 241 and 412 calves were assessed before and after the delivery of benchmarking reports, respectively, across enrolled farms.

At the pre-intervention baseline, the probability of calf dehydration was estimated at 60.6% (95% CI = [36.9, 80.1]) and 71.8% [53.6, 84.9] for intervention and control farms, respectively (*P* = 0.63; estimated OR = 0.60 [0.14, 2.62]). There was no evidence for any interaction (*P* = 0.14, 0.18) nor a main effect of benchmark reporting intervention (*P* = 0.25) and timing of health assessment (*P* = 0.73) on the probability of calf dehydration. The Bonferroni-adjusted pairwise comparisons showed the probability of dehydration for surplus calves from intervention farms was decreased (*P* = 0.04; estimated OR = 0.19 [0.04, 0.90]) compared with that of control farms following benchmark reporting (Figure 2). Relative to its baseline, intervention farms showed no decrease in calf dehydration after report delivery (*P* = 0.15; estimated OR = 0.38, 95% CI = 0.10, 1.39).

**Figure 2:**
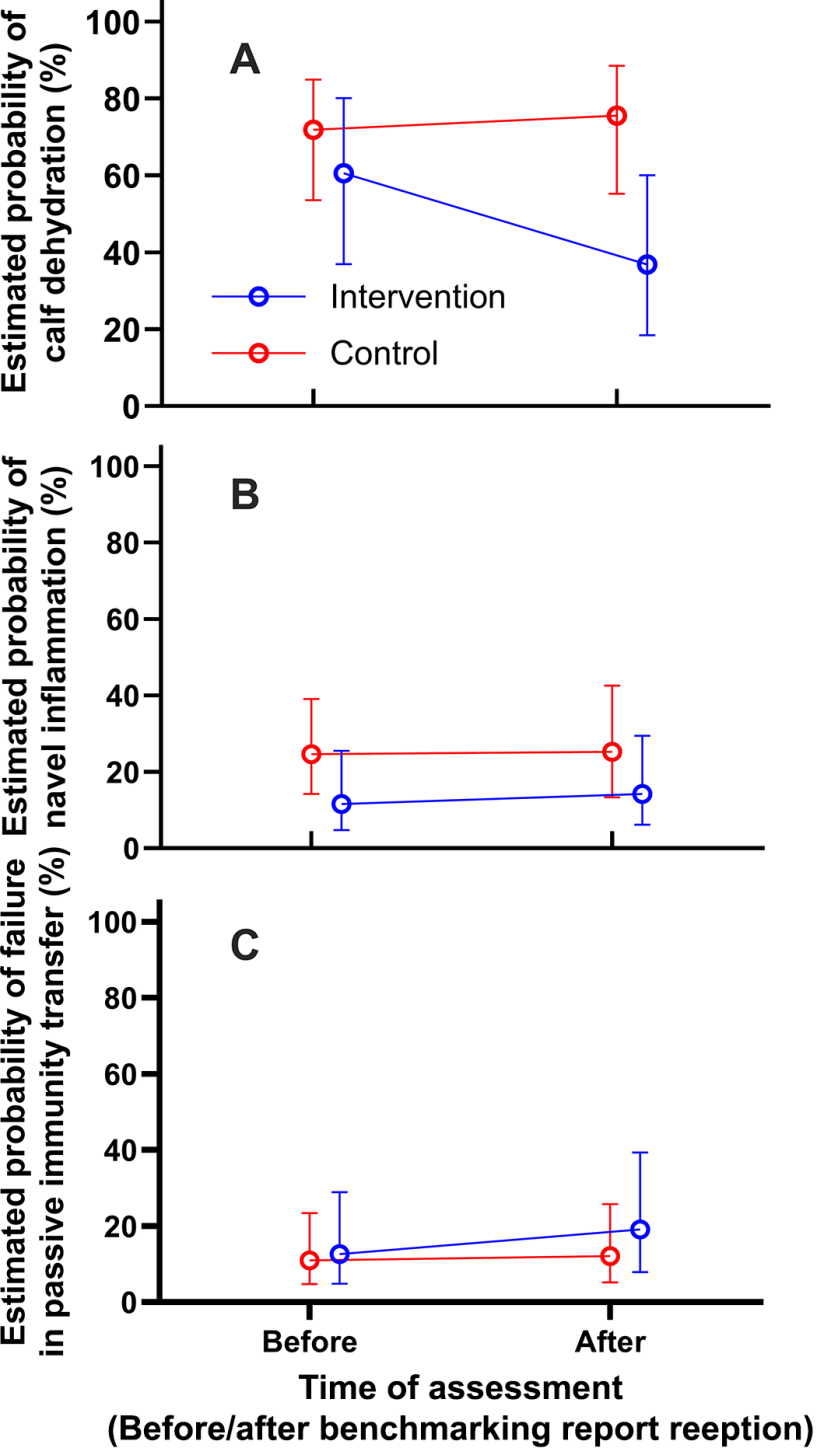
Estimated probability of dehydration (A), navel inflammation (B), and failed transfer of passive immunity (FTPI; C) in surplus dairy calves for intervention and control farms before and following delivery of benchmarking reports. Calf hydration was assessed using the skin tent test and a 4-point scale (Garcia et al., 2022), and navel health was assessed based on the width of umbilical cords using a 4-point scale (Pempek et al., 2017). Calves that received a dehydration or navel inflammation score of 1 to 3 were considered positive for corresponding signs, whereas those that received a score of 0 were labeled as negative. For FTPI, total serum protein concentrations were dichotomized using a cutoff of 5.1 g/dL (TSP ≥5.1 g/dL: FTPI absent; TSP <5.1 g/dL: FTPI present (Lombard et al., 2020). Whiskers represent 95% CI. No interaction of intervention by time relative to delivery of benchmarking report (*P* > 0.05).

The baseline probability of calf navel inflammation was estimated at 11.6% [4.7%, 25.5%] and 24.6% [14.3%, 39.1%] for intervention and control farms, respectively (*P* = 0.22; estimated OR = 0.40 [0.10, 1.16]). There was no evidence for any interaction (*P* = 0.77, 0.34), nor any main effects of benchmark reporting intervention or timing (*P* = 0.73 and *P* = 0.94, respectively) on the probability of navel inflammation. Following report delivery, there was no evidence supporting differences in the odds of a navel inflammation diagnosis between intervention and control farms (*P* = 0.38; estimated OR = 0.49 [0.12, 2.06]) after the delivery of benchmarking reports, nor intervention farms between 2 health assessment times (*P* = 0.87; estimated OR = 1.26 [0.37, 4.30]; Figure 2).

The probability for FTPI for calves from intervention and control farms was estimated as 12.6% (4.9, 29.0) and 11.0% (4.8, 23.4), respectively, at the baseline (*P* = 0.97; estimated OR = 1.17 [0.24, 5.76]). There was no evidence for any interaction (*P* = 0.63, 0.76) nor any main effects of benchmarking reporting intervention or timing (*P* = 0.80 and *P* = 0.85, respectively) on the probability of FTPI. The difference in odds of FTPI among calves from intervention versus control farms after report delivery was not supported by statistical evidence (*P* = 0.66, estimated OR = 1.72 [0.35, 8.48]) or intervention farms relative to their baseline (*P* = 0.60; estimated OR = 1.64 [0.44, 6.16]).

Calves with dehydration, navel inflammation, and low TSP were more likely to have diarrhea and an increased risk of morbidity and mortality (Renaud et al., 2018; Wilson et al., 2020). Access to calf performance data has been reported to motivate and encourage dairy producers to improve on-farm calf management and care practices (Atkinson et al., 2017). In addition, calf benchmarking information and peer comparisons have been proposed as a means to promote cooperation among herd veterinarians, farm managers, and calf caretakers to improve results (Sumner et al., 2018, 2020). Our results suggest using benchmarking reports may motivate producers to make improvements in preventing calf dehydration (e.g., water access or feeding before transportation), but may not be enough to make changes in navel health and colostrum feeding practices. That is, producers may be more willing to improve calf water intake in response to benchmarking reports, as this is straightforward, less costly, and less burdensome to farm workers than navel care and colostrum management. Still, delivering benchmarking reports covering comprehensive calf health metrics may allow dairy producers to thoroughly review their on-farm calf care practices and identify additional opportunities for improvement.

In addition to on-farm water availability, the distance and time of transportation from the source dairy farms to calf dealers have been associated with calf dehydration (Knowles et al., 1997). Transportation and care practices of the calf dealer were unlikely to be major drivers of calf dehydration observed in this study because calves were delivered from farms to neighboring calf dealers and assessed shortly upon arrival without receiving water, milk, or feed. Nevertheless, the effect of early-life transportation and management practices of calf dealers and auctions should be considered in future studies with a substantial time-lapse between calf delivery and health assessment.

The marginal effect of receiving benchmarking reports on improving calf navel health and passive transfer may be attributed to several reasons. First, not all dairy producers were proactive and could be motivated solely by accessing benchmarking resources to change calf management practices (Wilson et al., 2023). Such barriers included the lack of (1) resources (e.g., time, money, space), (2) responsibility for providing calf care, and (3) apparent improvements after previous management changes. Additionally, although the delivery was done in person by study personnel, the intervention may have had a larger impact if we included the herd veterinarians or major advisors (or both) for calf management at the benchmarking report delivery meetings. Herd veterinarians are known for their critical role to dairy producers in presenting benchmarking results, providing explicit recommendations, and advocating the implementation of changes (Sumner et al., 2020). Such an effect must be further investigated along with potential barriers preventing dairy producers from improving calf care practices.

Several limitations must be considered when interpreting the results of the current study. First, the amount of true replication was small, as the experimental unit for assessing the effect of benchmarking report delivery was defined by farms rather than by individual calves, which served as technical replicates or subsamples. Additionally, the data were unbalanced as the number of calves assessed after the report delivery was small for several farms (e.g., farms 3, 12, 13 in Table 1), which limited the use of farm-level models that assigned equal weights of estimation to farms. Furthermore, 3 farms were added to the study post hoc and not subjected to random assignment to treatment. Although such limitation occurs in studies with a small size of experimental units, the lack of randomization may lead to inadvertent confounding biases with the estimate of the effect of intervention. Furthermore, changes in calf care practices by dairy farmers were not directly measured. Further study is warranted to understand how benchmarking influences decision-making among producers with regard to the care of surplus calves.

This study suggests that delivering benchmarking reports to dairy producers may help prevent dehydration in subsequent cohorts of surplus calves. To the best of our knowledge, this is the first investigation of the use of benchmarking reports in motivating US dairy producers to improve care practices for surplus calves. These findings may identify opportunities to improve on-farm calf care practices by informing dairy producers about the postmarketing performance of calves from their farms.
